# Megestrol acetate in the management of cancer cachexia: a prospective quasi-experimental study focusing on body composition and patient-reported outcomes

**DOI:** 10.3389/fnut.2026.1780653

**Published:** 2026-04-21

**Authors:** Xiaoting Zhong, Boyang Kuang, Kuan Yang, Huang Zhou, Chenxi Yuan, Xiangcai Wang, Hongmei Chen, Xiaoli Zeng, An Li, Weiling Lai, Xiaoqing Guo, Qi Wang, Jianming Ye

**Affiliations:** 1 Department of Oncology, The First Affiliated Hospital of Gannan Medical University, Ganzhou, Jiangxi, China; 2 Gannan Medical University, Ganzhou, Jiangxi, China; 3 Jiangxi “Flagship” Oncology Department of Synergy for Chinese and Western Medicine, Ganzhou, Jiangxi, China; 4 Jiangxi Provincial Unit for Clinical Key Oncology Specialty Development, Ganzhou, Jiangxi, China; 5 Jiangxi Clinical Research Center for Cancer, Ganzhou, Jiangxi, China; 6 Department of Oncology, The First Affiliated Hospital, Jinan University, Guangzhou, China

**Keywords:** cancer cachexia, cancer-related fatigue, megestrol acetate, nutritional status, quality of life

## Abstract

**Background:**

The objective of this study is to investigate the efficacy of megestrol acetate (MA) in treating cancer cachexia from multiple dimensions.

**Methods:**

In this prospective, non-randomized study, 97 patients with cancer cachexia were allocated to either a control group (*n* = 33, regular diet) or an MA group (*n* = 64, regular diet plus MA 320 mg/day) for 2 months. The primary endpoints were nutritional indices, including weight, BMI, total skeletal muscle mass, and fat mass. Secondary endpoints included inflammatory markers (CRP, IL-6, TNF-*α*), immune parameters (CD4^+^, CD8^+^ T cells), cancer-related fatigue (assessed by the Cancer Fatigue Scale), and quality of life (QOL).

**Results:**

Compared to baseline, the MA group exhibited significant improvements in body weight, BMI, fat mass, prealbumin (PA), albumin (ALB), and hemoglobin (Hb), coupled with a significant reduction in IL-6 levels and all domains of cancer-related fatigue (somatic, cognitive, affective, and total). Between-group analyses demonstrated that the MA group achieved significantly greater improvements in weight, BMI, fat mass, PA, ALB, and Hb. Skeletal muscle mass was maintained in the MA group, whereas the control group experienced a significant loss, resulting in a significant between-group difference. Furthermore, the MA group showed markedly greater reductions in all fatigue domain scores and a more substantial improvement in QOL. No significant between-group differences were observed for most inflammatory or immune markers. The intervention was well-tolerated with no reported drug-related adverse events.

**Conclusion:**

MA significantly improves nutritional status and ameliorates cancer-related fatigue, thereby enhancing the quality of life in patients with cancer cachexia. Our findings provide robust evidence supporting the multi-dimensional benefits of MA in cachexia management, extending beyond mere weight gain to include muscle mass preservation and patient-centered symptom relief.

## 1 Introduction

Cancer cachexia represents a devastating paraneoplastic syndrome, defined by progressive loss of skeletal muscle mass (with or without loss of fat mass), which leads to functional impairment and poor prognosis (1). It affects a majority of patients with advanced cancer and is directly responsible for a significant proportion of cancer-related deaths (2). The clinical trajectory of cachexia is marked by anorexia, weight loss, severe fatigue, and a diminished response to anti-cancer therapies (3, 4). Underlying these symptoms is a state of chronic, systemic inflammation driven by pro-inflammatory cytokines such as TNF-*α*, IL-6, and CRP, which promote a metabolic shift favoring catabolism over anabolism (5).

Current management strategies for cancer cachexia remain suboptimal, with no universally accepted standard pharmacological therapy. Megestrol acetate (MA), a synthetic progestin, has been widely used for its appetite-stimulating and weight-gaining effects in this context (6, 7). However, weight alone is a crude metric that fails to distinguish between gains in fat mass and the critical preservation of lean body mass (8). The efficacy of MA in specifically counteracting the core pathology of cachexia-skeletal muscle wasting is not well-established, with existing studies yielding inconsistent results (9, 10). Furthermore, while fatigue is a primary concern for patients, comprehensive data on the impact of MA on multi-dimensional cancer-related fatigue and overall quality of life are scarce.

To address these gaps, we conducted this prospective study to provide a holistic evaluation of MA in cancer cachexia. Utilizing precise bioelectrical impedance analysis for body composition assessment, we aimed to delineate its effects on skeletal muscle and fat mass independently. Additionally, we systematically evaluated its influence on inflammatory status, immune function, and, crucially, patient-centric outcomes including cancer-related fatigue and quality of life.

## 2 Materials and methods

### 2.1 Study design and subjects

**Figure 1:**
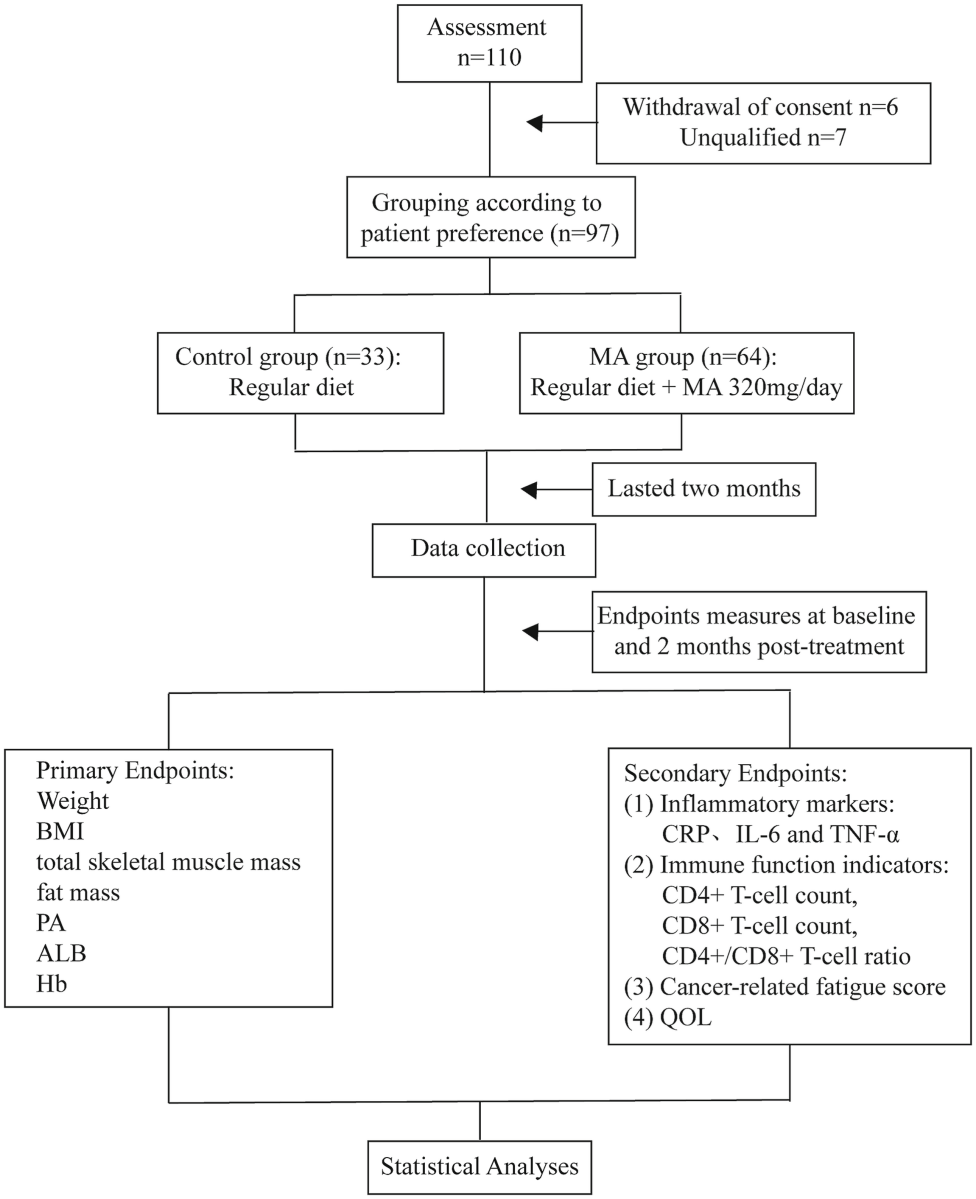
Study flowchart. A total of 110 patients meeting the diagnostic criteria for cancer cachexia were enrolled at the First Affiliated Hospital of Gannan Medical University between December 2023 and October 2024. Thirteen cases were excluded during screening, resulting in 97 patients being included. Participants were randomly assigned according to their preference to either the control group (*n* = 33) or the MA group (*n* = 64).

This prospective, non-randomized, quasi-experimental study was conducted at the First Affiliated Hospital of Gannan Medical University between December 2023 and October 2024. This design was chosen to more closely reflect actual clinical practice and mitigate dropouts associated with mandatory randomization. The study protocol was approved by the hospital’s Ethics Committee (No: 22SC-2025284), and all participants provided written informed consent. A total of 110 patients with cancer cachexia were screened, and 97 were ultimately enrolled (Figure 1).

Group allocation was based on patient preference to enhance real-world feasibility and compliance, resulting in 33 patients in the control group and 64 in the MA group. The decision to use a preference-based allocation rather than a randomized controlled trial (RCT) was made for practical and ethical considerations in the context of clinical nutritional interventions. First, in our clinical setting, patients with cancer cachexia often have strong preferences regarding pharmacological interventions; pilot observations indicated that mandatory randomization would have led to high refusal and dropout rates (estimated >30%), potentially compromising the validity of an RCT more severely than a well-analyzed observational study. Second, this study was designed as a pragmatic, real-world investigation to evaluate MA’s effectiveness under conditions that reflect actual clinical practice, where patients’ treatment choices are often respected. Such quasi-experimental designs are commonly employed in cachexia research when RCTs are challenging to conduct. Third, given that MA is already widely used for appetite stimulation in cachexia, denying it to patients with strong preferences was considered ethically problematic, particularly in the absence of a universally accepted standard therapy. A generalized estimating equations (GEE) was employed to analyse inter- and intra-group differences in efficacy indicators.

### 2.2 Inclusion and exclusion criteria

Inclusion criteria: (1) histologically or cytologically confirmed malignancy; (2) diagnosis of cachexia according to Fearon’s criteria (6): excludes simple starvation, with weight loss exceeding 5% within 6 months, or BMI < 20 kg/m^2^ with weight loss exceeding 2%, or meets sarcopenia diagnostic criteria (skeletal muscle index of limbs <7.26 kg/m^2^ for males, <5.45 kg/m^2^ for females) with weight loss exceeding 2%; (3) good compliance; (4) an expected survival of >6 months.

Exclusion criteria include: (1) known allergy to MA; (2) severe cardiac, cerebral, hepatic, or renal dysfunction; (3) concurrent psychiatric disorder or inability to comply with medical instructions; (4) conditions preventing oral intake.

### 2.3 Intervention

The control group received a regular diet alongside their standard oncologic care. The MA group received oral MA (320 mg/day) in addition to a regular diet for 2 months. Both groups continued their planned anti-tumor treatments and necessary supportive care.

### 2.4 Outcomes assessment

Assessments were performed at baseline and after 2 months of intervention.

*Primary endpoints*: Nutritional status, including body weight, BMI, total skeletal muscle mass, fat mass (measured using the InBody S10 analyzer), and serum levels of prealbumin (PA), albumin (ALB), and hemoglobin (Hb). Body weight was recorded after patients had voided urine and defecated in the morning on an empty stomach. BMI was calculated as weight (kg) divided by height squared (m^2^). PA, ALB, and Hb were assessed as blood parameters; all subjects underwent venous blood sampling in the morning on an empty stomach prior to breakfast for testing. The InBody (S10) body composition analyser was employed to measure patients’ total skeletal muscle mass and fat mass. Prior to testing, subjects fasted for 2 h, abstained from drinking for 1 h, and emptied their bowels and bladder. Testing was conducted in accordance with the instrument’s operating manual, with data subsequently downloaded directly from the system upon completion.

*Secondary endpoints*: Inflammatory markers, immune function parameters, cancer-related fatigue, and quality of life. The inflammatory markers include: C-reactive protein (CRP), interleukin-6 (IL-6), and tumour necrosis factor-alpha (TNF-*α*). Serum IL-6 and TNF-α levels were measured using an enzyme-linked immunosorbent assay (ELISA). Immune function parameters include: CD4^+^ T lymphocyte count, CD8^+^ T lymphocyte count, and CD4^+^/CD8^+^ T lymphocyte ratio, measured using flow cytometry. The Cancer Caused Fatigue Score (CFS), developed by Okuyama et al. (11), was employed to assess cancer caused fatigue symptoms, encompassing Somatic fatigue (7 items), Cognitive fatigue (4 items), and Affective fatigue (4 items). This scale is scored on a severity scale of 1 to 5, yielding a total score ranging from 0 to 60, where a higher score indicates more severe fatigue. The Chinese Cancer Patient Quality of Life (QOL) Rating Scale (12), established in 1990, was employed to assess patients’ quality of life before and after treatment. Based on QOL scores, evaluations encompassed appetite, mental state, sleep patterns, fatigue, pain symptoms, alongside social understanding and cooperation, patients’ comprehension of their cancer, attitudes towards treatment, daily living activities, adverse reactions, and facial expressions. Each item was scored from 1 to 5, with a maximum total score of 60. Higher scores indicated better quality of life.

*Safety evaluation*: Adverse events were monitored and graded according to CTCAE v5.0.

All 97 enrolled patients completed the 2-month follow-up, with no dropouts during the study period. However, missing data were present for some laboratory and body composition parameters due to occasional insufficient blood sample volume or failure to complete bioelectrical impedance analysis. Specifically, missing rates were as follows: prealbumin 9.3%, CRP 12.4%, IL-6 17.5%, TNF-*α* 15.5%, CD4^+^ and CD8^+^ T lymphocyte count 13.4%, CD4^+^/CD8^+^ T lymphocyte ratio 15.5%, skeletal muscle mass 8.2%, and fat mass 8.2%. No missing data occurred for weight, BMI, Hb, ALB, fatigue scores, or quality of life. All missing values were due to logistical reasons (e.g., insufficient sample volume, equipment unavailability) rather than assay range limitations, as no values were excluded for falling outside ELISA detection limits. The GEE approach used for primary analyses accommodates missing data under the missing completely at random assumption, and all available data were included in the analyses.

### 2.5 Sample size calculation

The sample size was calculated based on the primary outcome of change in total skeletal muscle mass. Pre-trial data assumed a change of −1.5 ± 2.0 kg in the control group and −0.5 ± 2.0 kg in the MA group. Using PASS 15.0 software for comparing two independent means (*α* = 0.05, two-tailed; 1-*β* = 0.80), a minimum of 44 patients per group (total *N* = 88) was required. Accounting for the non-randomized design with an expected 2:1 (MA: control) enrolment ratio to enhance MA group recruitment, the total sample size was adjusted to 93. Factoring in a 10% dropout rate, the final planned sample size was 97 (MA: 65, control: 32). The final analysis included 64 MA and 33 control patients, aligning with the plan.

### 2.6 Statistical analyses

Continuous normally distributed data are presented as mean ± standard deviation and compared using *t*-tests (two groups) or one-way ANOVA (multiple groups). Non-normally distributed data are presented as median [P25, P75] and compared using the rank-sum test. Categorical data are presented as frequencies (%), compared using chi-square or Fisher’s exact tests. For longitudinal/repeated-measures data, the R language `geepack` package was used to perform Generalized Estimating Equations (GEE). An ‘exchangeable’ correlation structure was specified. Baseline measurements (which showed imbalance, e.g., tumor type), age, gender, and tumor type were included as fixed-effect covariates in the models to control for potential confounding. The models estimated least squares means and standard errors for different groups at different time points. Within-group comparisons were also performed using GEE. Visualization was achieved using `ggplot2`. All analyses were performed using R software (version 4.3.3). A two-sided *p*-value < 0.05 was considered statistically significant.

## 3 Results

### 3.1 Clinical characteristics

As detailed in Table 1, the baseline characteristics of the two groups were largely comparable. However, a significant difference was noted in tumor type distribution (*p* = 0.037), with a higher proportion of gastrointestinal tumors in the MA group. This baseline imbalance was statistically accounted for in the subsequent GEE models.

**Table 1:** Baseline characteristics of the 97 enrolled patients.

Characteristic	Overall (*n* = 97)	Control group (*n* = 33)	MA group (*n* = 64)	*p*-value
Age, mean ± SD	61.68 ± 9.56	64.06 ± 8.69	60.45 ± 9.82	0.068
Gender, *n* (%)				
Male	64 (65.98)	23 (69.70)	41 (64.06)	
Female	33 (34.02)	10 (30.30)	23 (35.94)	0.579
Tumour type, *n* (%)				0.037
Gastrointestinal tumours	51 (52.58)	6 (18.18)	25 (39.06)	
Non-gastrointestinal tumours	46 (47.42)	27 (81.82)	39 (60.94)	
Weight (kg)	50.07 ± 8.81	49.97 ± 8.89	50.12 ± 8.85	0.938
BMI (kg/m^2^)	19.25 ± 2.51	19.13 ± 2.58	19.32 ± 2.49	0.729
Skeletal muscle mass (kg)	21.68 ± 4.67	21.13 ± 4.60	21.97 ± 4.72	0.410
Fat mass (kg)	9.46 ± 4.18	9.73 ± 3.63	9.31 ± 4.46	0.646
PA (mg/L)	165.60 ± 62.45	169.00 ± 65.59	163.85 ± 61.22	0.702
ALB (g/L)	36.34 ± 4.54	36.65 ± 4.38	36.18 ± 4.65	0.637
Hb (g/L)	114.03 ± 20.82	115.79 ± 22.55	113.12 ± 19.99	0.553
CRP (mg/L)	31.08 ± 44.62	36.31 ± 57.48	28.41 ± 36.68	0.418
IL-6 (pg/mL)	28.92 ± 58.63	33.70 ± 81.78	26.73 ± 45.00	0.612
TNF-*α* (pg/mL)	2.09 ± 1.34	1.81 ± 0.86	2.22 ± 1.49	0.183
CD4^+^ T cells (cells/μL)	463.83 ± 255.93	458.85 ± 281.53	466.08 ± 246.03	0.904
CD8^+^ T cells (cells/μL)	366.08 ± 232.31	316.22 ± 224.96	388.52 ± 233.91	0.181
CD4^+^/CD8^+^ T ratio	1.52 ± 0.78	1.68 ± 0.78	1.45 ± 0.77	0.202
Somatic fatigue score	13.04 ± 5.00	13.97 ± 4.88	12.56 ± 5.03	0.190
Cognitive fatigue score	4.70 ± 2.45	4.49 ± 2.11	4.81 ± 2.62	0.536
Affective fatigue score	6.72 ± 2.02	6.67 ± 1.86	6.75 ± 2.11	0.848
Total fatigue score	24.46 ± 7.98	25.12 ± 7.42	24.12 ± 8.29	0.563
QOL	37.00 ± 5.25	37.45 ± 5.21	36.77 ± 5.29	0.543

Footnotes for Table 1: Data are presented as mean ± standard deviation or *n* (%). *p*-values were calculated using independent *t*-test for continuous variables, Mann–Whitney *U* test for non-normally distributed variables, and chi-square test for categorical variables. Statistically significant difference (*p* < 0.05) is indicated in bold. BMI, body mass index; PA, prealbumin; ALB, albumin; Hb, hemoglobin; CRP, C-reactive protein; IL-6, interleukin-6; TNF-*α*, tumor necrosis factor-alpha; QOL, quality of life; SD, standard deviation.

During the two-month intervention, most patients received concurrent anticancer therapy. In the control group, 30/33 patients (90.9%) received active treatment (chemotherapy, targeted therapy, immunotherapy, radiotherapy, interventional therapy, or combinations); the remaining 3 (9.1%) received best supportive care only. In the MA group, 59/64 (92.2%) received active treatment, and 5 (7.8%) received best supportive care alone. Treatment modality distributions were comparable between groups. Detailed information is provided in Supplementary Table S1. To better characterize potential selection bias, we collected data on patients’ reasons for allocation preferences. Among the 64 patients in the MA group, the primary reasons for choosing MA were: desire for appetite stimulation (*n* = 38, 59.4%), recommendation from family or other patients (*n* = 12, 18.8%), previous positive experience with MA (*n* = 8, 12.5%), and reluctance to receive additional medication (*n* = 6, 9.4%). Among the 33 patients in the control group, the primary reasons for declining MA were: concerns about side effects (*n* = 14, 42.4%), preference for non-pharmacological management (*n* = 10, 30.3%), financial constraints (*n* = 6, 18.2%), and physician recommendation against MA (*n* = 3, 9.1%).

All 97 patients completed the 2-month follow-up, with no study withdrawals. Missing values for laboratory and body composition parameters ranged from 8.2 to 17.5% due to logistical reasons, as detailed in the Methods section. No missing data were observed for primary nutritional parameters (weight, BMI, ALB) or patient-reported outcomes (fatigue scores, QOL). All available data were included in the GEE analyses.

### 3.2 Primary outcomes: nutritional status

**Figure 2:**
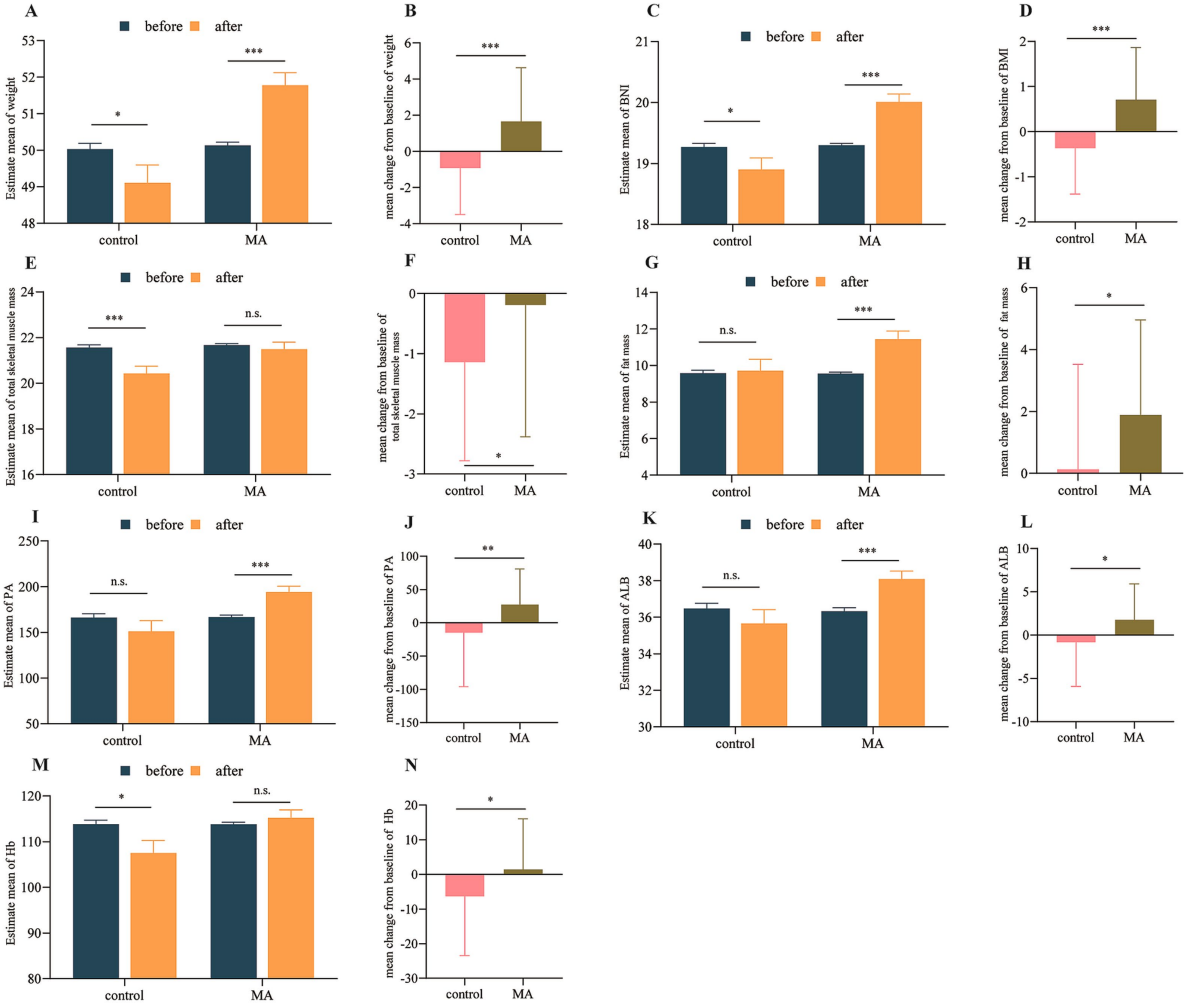
MA improved nutritional status in patients with cancer cachexia. **(A,C,E,G,I,K,M)** Within-group comparisons before and after intervention. **(B,D,F,H,J,L,N)** Between-group comparisons of changes after intervention. *p < 0.05; ** *p* < 0.01; *** *p* < 0.001; n.s., not significant.

Within-group comparisons revealed that the control group experienced significant declines in weight, BMI, skeletal muscle mass, and Hb after 2 months. In contrast, the MA group showed significant increases in weight, BMI, fat mass, PA, and ALB (Table 2, Figures 2A,C,E,G,I,K,M). However, changes in total skeletal muscle mass and Hb levels in the MA group were not statistically significant post-treatment (Table 2 and Figures 2E,M).

**Table 2:** Inter- and intra-group variability results using GEE [presenting least squares estimated mean (standard error)].

Characteristic	Control group (*n* = 33)			MA group (*n* = 64)			Control vs MA *p*-value
	Before	After	*p*	Before	After	*p*	
	Least squares estimated mean (standard error)			Least squares estimated mean (standard error)			
Weight (kg)	50.03 (0.16)	49.11 (0.49)	0.038	50.13 (0.09)	51.78 (0.34)	<0.001	<0.001
BMI (kg/m^2^)	19.27 (0.06)	18.90 (0.19)	0.039	19.30 (0.03)	20.01 (0.13)	<0.001	<0.001
Skeletal muscle mass (kg)	21.56 (0.12)	20.43 (0.32)	<0.001	21.67 (0.06)	21.49 (0.31)	0.546	0.032
Fat mass (kg)	9.58 (0.16)	9.71 (0.63)	0.844	9.55 (0.09)	11.43 (0.45)	<0.001	0.024
PA (mg/L)	166.19 (4.23)	151.33 (11.61)	0.287	166.92 (2.15)	194.14 (6.28)	<0.001	0.006
ALB (g/L)	36.48 (0.29)	35.66 (0.76)	0.351	36.33 (0.19)	38.09 (0.43)	0.001	0.011
Hb (g/L)	113.83 (0.89)	107.53 (2.72)	0.033	113.81 (0.46)	115.22 (1.75)	0.439	0.026
CRP (mg/L)	32.82 (4.52)	37.82 (11.63)	0.720	29.97 (2.31)	26.97 (4.84)	0.534	0.587
IL-6 (pg/mL)	27.76 (5.53)	24.87 (16.60)	0.890	26.65 (2.10)	17.63 (2.22)	0.009	0.761
TNF-*α* (pg/mL)	2.00 (0.08)	1.83 (0.15)	0.287	2.15 (0.07)	1.77 (0.21)	0.102	0.486
CD4^+^ T cells (cells/μL)	472.00 (13.51)	454.77 (48.53)	0.747	470.29 (18.87)	543.17 (41.60)	0.047	0.167
CD8^+^ T cells (cells/μL)	359.19 (13.25)	422.73 (80.25)	0.417	371.14 (17.50)	424.41 (40.29)	0.123	0.904
CD4^+^/CD8^+^ T ratio	1.48 (0.04)	1.43 (0.20)	0.815	1.50 (0.02)	1.43 (0.08)	0.434	0.939
Somatic fatigue score	12.98 (0.25)	13.25 (0.50)	0.593	12.84 (0.16)	9.17 (0.45)	<0.001	<0.001
Cognitive fatigue score	4.54 (0.14)	5.24 (0.26)	0.011	4.73 (0.12)	3.54 (0.23)	<0.001	<0.001
Affective fatigue score	6.58 (0.15)	6.30 (0.40)	0.528	6.69 (0.10)	4.41 (0.20)	<0.001	<0.001
Total fatigue score	24.18 (0.42)	24.88 (0.92)	0.455	24.23 (0.29)	17.09 (0.66)	<0.001	<0.001
QOL	37.31 (0.28)	37.74 (0.69)	0.566	37.07 (0.16)	41.57 (0.55)	<0.001	<0.001

Footnotes for Table 2: Data are presented as least squares estimated mean with standard error derived from generalized estimating equations (GEE) with an exchangeable correlation structure. Within-group comparisons (before vs. after) and between-group comparisons (MA group vs. control group) were performed using GEE. All models were adjusted for age, gender, and tumor type to control for potential confounding. Statistically significant differences (*p* < 0.05). PA, prealbumin; ALB, albumin; Hb, hemoglobin; CRP, C-reactive protein; IL-6, interleukin-6; TNF-*α*, tumor necrosis factor-alpha; QOL, quality of life; GEE, generalized estimating equations.

Critically, between-group comparisons demonstrated that the MA group had significantly superior outcomes compared to the control group. Patients receiving MA exhibited significantly greater increases in weight, BMI, fat mass, PA, ALB, and Hb (all *p* < 0.05, Figures 2B,D,H,J,L,N). Most notably, skeletal muscle mass was maintained in the MA group (within-group change: *p* = 0.546), whereas the control group experienced a significant loss (*p* < 0.001), leading to a significant between-group difference (*p* = 0.032, Figure 2F). Individual changes and distribution of nutritional indicators before and after intervention as shown in Supplementary Figure S1.

### 3.3 Secondary outcomes

**Figure 3:**
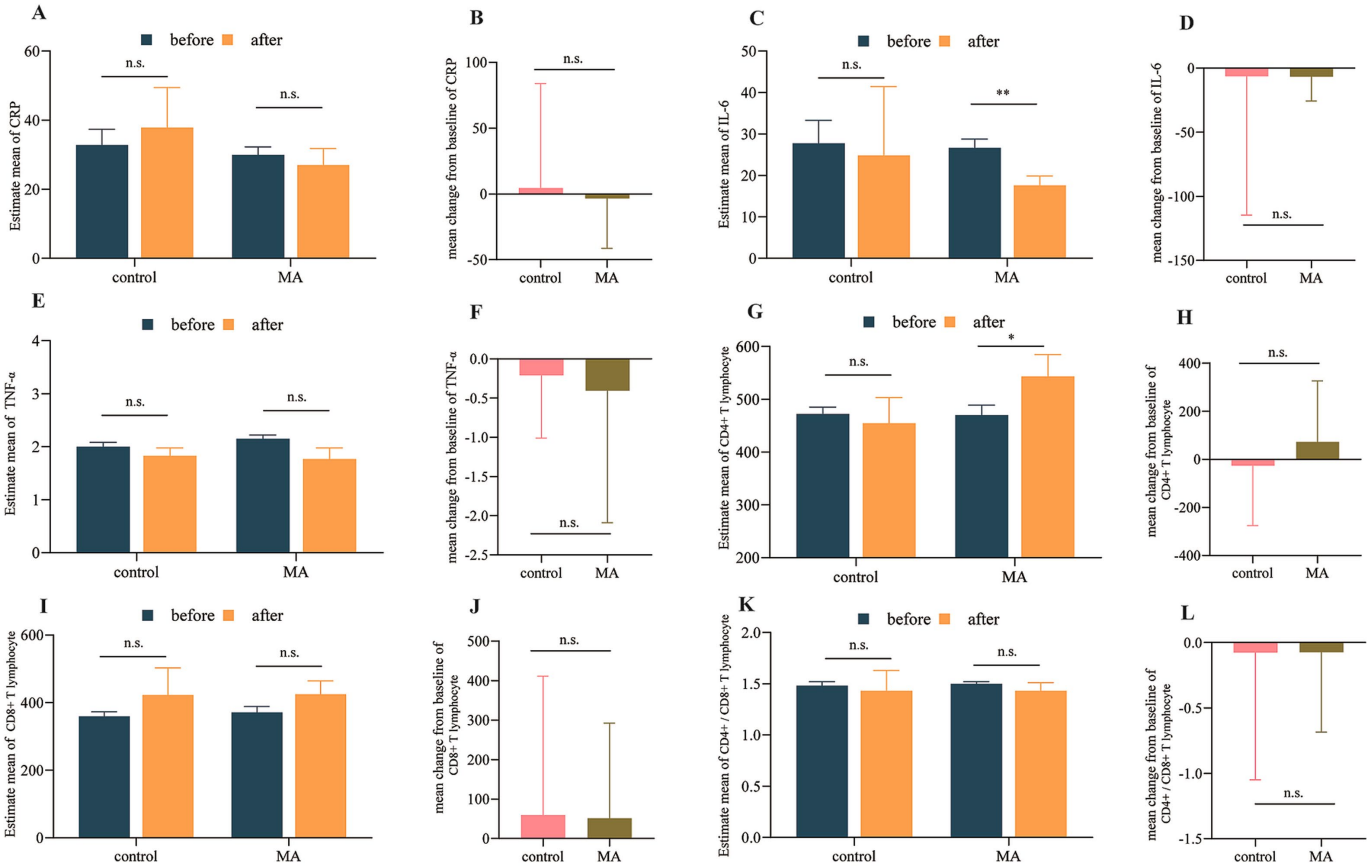
MA showed no significant effect on inflammatory levels or immune function in patients with cancer cachexia. **(A,C,E,G,I,K)** Within-group comparisons before and after intervention. **(B,D,F,H,J,L)** Between-group comparisons of changes after intervention. *p < 0.05; **p < 0.01; ***p < 0.001; n.s., not significant.

*Inflammation and immunity*: While the MA group showed a significant within-group reduction in IL-6 (*p* = 0.009), the between-group difference for the change in IL-6 was not significant (*p* = 0.761). This was due in part to a numerical, though non-significant, decrease in IL-6 in the control group as well. No significant between-group differences were observed for changes in CRP or TNF-*α* (Figures 3A–F). Similarly, although CD4^+^ T-cell count increased within the MA group, the between-group changes in CD4^+^ and CD8^+^ T-cell counts and their ratio were not statistically significant (Figures 3G–L). Individual changes and distribution of inflammatory and immune parameters before and after intervention as shown in Supplementary Figure S2.

**Figure 4:**
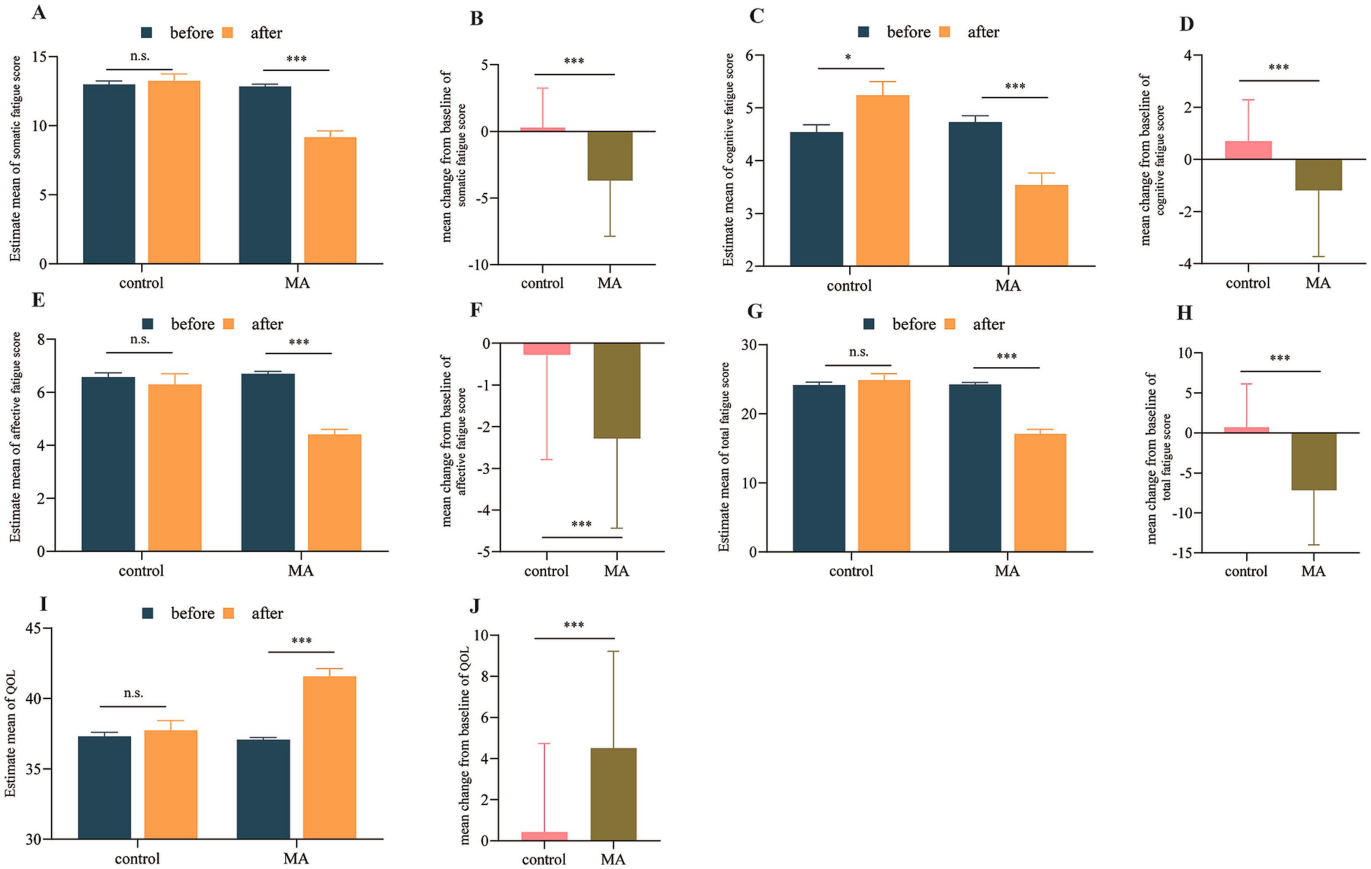
MA ameliorated fatigue and enhanced quality of life in patients with cancer cachexia. **(A,C,E,G,I)** Within-group comparisons before and after intervention. **(B,D,F,H,J)** Between-group comparisons of changes after intervention. *p < 0.05; **p < 0.01; ***p < 0.001; n.s., not significant.

*Fatigue and quality of life*: The MA group demonstrated significant and profound reductions in all fatigue domains (somatic, cognitive, affective) and the total fatigue score from baseline (all *p* < 0.001). Compared to the control group, the reductions in fatigue scores were significantly greater in the MA group (all *p* < 0.01, Figure 4A–H). Consistently, the improvement in QOL was significantly more substantial in the MA group than in the control group (*p* < 0.001, Figures 4I,J). Individual changes and distribution of fatigue scores and quality of life before and after intervention as shown in Supplementary Figure S3.

### 3.4 Safety outcomes

No MA-related adverse events were reported during the 2-month study period.

## 4 Discussion

Cancer cachexia has a major impact on patients ‘quality of life and survival, improving patients’ nutritional status is pivotal in the management of cancer cachexia (13). This prospective study provides compelling evidence that megestrol acetate offers multi-faceted benefits in the management of cancer cachexia. Our findings confirm its established role in promoting weight gain and improving nutritional biomarkers, but more importantly, they shed new light on its potential to modify body composition and profoundly impact patient-reported symptoms.

The efficacy of megestrol acetate in ameliorating nutritional status and stimulating weight gain in cancer cachexia is supported by prior research. Our findings corroborate existing literature, demonstrating that a 2-month MA regimen resulted in significant increases in body weight and BMI compared to the control group. Notably, we also observed substantial improvements in key nutritional biomarkers, including prealbumin, albumin, and hemoglobin, suggesting a broad enhancement of nutritional status that extends beyond simple weight gain. This aligns with the results of a comprehensive meta-analysis by Ruiz-García et al. (7) which consolidated evidence from 38 randomized controlled trials (*n* = 4,304) and concluded that MA significantly improved appetite and promoted weight gain compared to placebo (RR = 2.36, 95% CI: 1.00–3.71). The utility of MA is not confined to adults; its beneficial effects have been documented in pediatric populations as well. For instance, Cuvelier et al. (13) demonstrated that MA significantly improved body weight, BMI, and mid-upper arm circumference in children with cancer cachexia. The collective evidence, including our data, underscores that MA intervention facilitates weight gain and appears to enhance overall nutritional status, potentially through the promotion of protein anabolic metabolism (14).

A key and novel finding of our study is that skeletal muscle mass was maintained in the MA group (within-group change: *p* = 0.546), whereas the control group experienced a significant loss (*p* < 0.001), resulting in a significant between-group difference (*p* = 0.032). While weight gain in cachexia is often assumed to be primarily adipose tissue, our data suggest that MA may exert a modest but clinically meaningful muscle-sparing effect, as it prevented the muscle loss observed in the control group. This finding is crucial because skeletal muscle wasting is the hallmark of cachexia and a strong predictor of mortality and functional decline (8). In an animal model study using MA and tumour-bearing rats (Yoshida AH-130) (15, 16), researchers observed increased intake of skeletal muscle metabolic substrates (16) and enhanced muscle mass in rats, indicating MA possesses muscle anabolic effects (17). Frost et al. (16) observed that MA may promote skeletal muscle growth by enhancing the biochemical availability of IGF-1 in patients with advanced breast cancer. The underlying mechanism remains to be fully elucidated but may involve the modulation of insulin-like growth factor (IGF-1) pathways or a reduction in catabolic drivers, as suggested by the downward trend in inflammatory cytokines (9, 10). The significant increase in fat mass aligns with MA’s known metabolic effects and contributes to overall energy reserves.

Chronic systemic inflammation is a cornerstone of cancer cachexia pathophysiology, with cytokines such as CRP, IL-6, and TNF-*α* acting as key drivers of metabolic dysfunction and catabolism (4). Studies have demonstrated (18, 19) that MA can reduce the production and release of pro-inflammatory cytokines (such as IL-1, IL-6, and TNF-*α*), thereby alleviating cancer cachexia symptoms to some extent. This confirmed that MA may possess anti-inflammatory properties. However, The limited observed effects on systemic inflammatory markers (CRP, TNF-*α*) and most immune parameters are consistent with several previous reports (12, 20). This suggests that the primary benefits of MA at a dose of 320 mg/day may not be mediated through potent, broad-spectrum anti-inflammatory or immunomodulatory actions. Instead, its efficacy appears to stem predominantly from its central appetite-stimulating and subsequent metabolic effects. The single-center, non-randomized design and the relatively short intervention period are limitations of our study, which may affect the generalizability of the findings and the power to detect more subtle changes in inflammation and immunity. The IL-6 results warrant further discussion. Although a significant within-group reduction was observed in the MA group, this did not translate into a significant between-group difference, primarily because the control group also showed a numerical decrease in IL-6, albeit non-significant. Several factors may explain this pattern. First, the high inter-subject variability in IL-6 levels (as reflected by the large standard deviations in Table 1) may have limited the statistical power to detect a between-group difference, particularly given the relatively small sample size of the control group (*n* = 33). Second, the study was originally powered for the primary outcome of skeletal muscle mass, not for secondary inflammatory endpoints; thus, it may have been underpowered to detect modest differences in IL-6 between groups. These considerations highlight the need for future studies with larger sample sizes and dedicated power calculations for inflammatory endpoints to more definitively evaluate the anti-inflammatory effects of MA.

The interplay between cachexia and immune dysfunction is complex and critical, given the role of a competent immune system in antitumour surveillance (21, 22). T lymphocytes are central to adaptive immunity, with CD4^+^ T cells orchestrating immune responses and CD8^+^ T cells executing cytotoxic functions (23, 24) The CD4^+^/CD8^+^ ratio serves as a marker of immune homeostasis (25). In this study, the MA group exhibited a significant increase in CD4^+^ T cell counts from baseline, indicating a potential immunomodulatory benefit. However, this change was not significant relative to the control group, and no significant effects were observed on CD8^+^ T cell counts or the CD4^+^/CD8^+^ ratio. This could be attributable to the limited sample size and the relatively short intervention period. Furthermore, the functional characterization of T-cell subpopulations (e.g., naive, memory, exhausted) was beyond the scope of this study, presenting a valuable avenue for future research to fully delineate the immunologic impact of MA.

Cancer-related fatigue (CRF) is a pervasive and debilitating symptom, affecting approximately 65% of patients (26), characterized by profound exhaustion that is disproportionate to recent activity and unrelieved by rest. A pivotal and highly significant finding of our study is the robust and comprehensive amelioration of CRF across all domains—somatic, cognitive, and affective—following MA treatment. Fatigue is one of the most distressing symptoms for cancer patients, and its improvement is a major therapeutic goal. The mechanisms underlying this benefit are likely multifactorial. We postulate that the synergy of improved overall nutritional status, enhanced energy availability from gains in both fat mass and preserved muscle mass, correction of anemia (reflected by increased Hb levels), and a potential modest modulation of the pro-inflammatory milieu collectively contributed to the observed fatigue relief (11). This dramatic alleviation of fatigue directly translated into a statistically significant and clinically meaningful improvement in quality of life, highlighting a profound patient-centric benefit of MA therapy that extends beyond conventional biomedical endpoints.

This study prioritized patient compliance and the practical feasibility of clinical nutritional interventions. Although a randomized controlled design was not employed, we utilized GEE to address the non-randomized allocation and baseline imbalances, such as the unequal distribution of gastrointestinal tumors. By incorporating these baseline variables as covariates into the model, the study effectively corrected for baseline differences in outcome measures.

Concurrently, the model adjusted for baseline levels of age, gender, and tumour type corresponding to outcome measures, thereby balancing potential confounding biases in the findings. In non-randomised clinical studies, the GEE can effectively addresses latent confounding biases and inter-group disparities through covariate adjustment, enhancing the reliability of causal inferences. During the study, insufficient follow-up frequency may have resulted in missing data for certain outcome measures. However, the GEE employed primarily focuses on the average effect across the population. It accounts for the correlation between repeated measurements through a working correlation matrix and utilises generalised estimating equations for parameter estimation. Regarding missing data handling, the GEE defaults to analysis based on observed data, assuming ‘completely at random’ (CAR) missingness. This approach yields unbiased estimates, further enhancing the reliability of the analytical results.

Several limitations of this study should be acknowledged. Most importantly, the non-randomized, preference-based allocation introduces a substantial risk of selection bias. Patients who chose to receive MA may have differed from those who declined in unmeasured factors such as motivation, baseline symptom burden, or prognosis, which could influence outcomes independently of the intervention. Although we attempted to control for measured confounders using GEE and stratified analyses, such statistical adjustments cannot fully account for bias from unmeasured confounders inherent in non-randomized designs. Therefore, our findings should be interpreted with caution, and the observed benefits of MA require confirmation in well-designed randomized controlled trials. First, the follow-up duration was limited to 2 months. Cancer-related fatigue and quality of life are dynamic and may fluctuate over the course of the disease, particularly in patients with gastrointestinal malignancies who often face chronic nutritional challenges that may be influenced by both the tumor itself and ongoing anticancer therapies. The relatively short observation period may not fully capture the longitudinal trajectory of these patient-reported outcomes.

Second, our study included patients with various primary tumor sites, each with distinct disease timelines, treatment regimens, and prognostic trajectories. Although we performed stratified analysis by tumor type and adjusted for cancer site in the GEE models, the heterogeneity of the study population may still limit the generalizability of our conclusions. Future studies with longer follow-up periods and more homogeneous patient cohorts are warranted to further validate the sustained benefits of MA across different cancer types and treatment phases. Third, while no patients were lost to follow-up, missing data were present for some laboratory and body composition parameters (ranging from 8.2 to 17.5%), primarily due to logistical constraints such as insufficient blood sample volume or equipment availability. These missing values were assumed to be missing completely at random and were handled using GEE, which provides valid estimates under this assumption. Nevertheless, the possibility of non-random missingness cannot be entirely excluded. We acknowledge that statistical adjustments (GEE with covariates) cannot fully eliminate bias from unmeasured confounders. However, we attempted to minimize bias by adjusting for all measured baseline imbalances (age, gender, tumor type), and using GEE which accounts for within-patient correlation and provides robust estimates under the missing-at-random assumption. Nevertheless, residual confounding remains possible.

Additionally, the sample size calculation was based on an assumed change in skeletal muscle mass (loss of 0.5 kg in the control group vs. no loss in the MA group) that was not fully realized, as the MA group maintained muscle mass while the control group lost 1.13 kg. This discrepancy suggests the study may have been underpowered to detect smaller, but still clinically meaningful, differences in its primary outcome. Furthermore, the study was powered primarily for the primary outcome of skeletal muscle mass. For secondary endpoints such as IL-6 and other inflammatory markers, the sample size may have been insufficient to detect modest between-group differences, especially given the high inter-subject variability observed in these measures. This potential underpowering for secondary outcomes should be considered when interpreting the null findings for inflammatory and immune parameters. Taken together, these considerations underscore the need for larger confirmatory studies to validate the findings and more precisely estimate the effects of MA on both primary and secondary outcomes.

## 5 Conclusion

In conclusion, our results affirm that megestrol acetate is an effective therapeutic option for cancer cachexia. It not only improves traditional nutritional parameters but also appears to help maintain skeletal muscle mass, as evidenced by the preservation of muscle mass in the MA group compared to significant loss in the control group. MA also provides remarkable relief from cancer-related fatigue, thereby significantly improving patients’ quality of life. These multi-dimensional benefits strengthen the evidence base for MA’s use in clinical practice and highlight the importance of evaluating body composition and patient-reported outcomes in future cachexia research.

## Data Availability

The original contributions presented in the study are included in the article/Supplementary material, further inquiries can be directed to the corresponding authors.
