# Supplementary figures and images for: Megestrol acetate in the management of cancer cachexia: a prospective quasi-experimental study focusing on body composition and patient-reported outcomes
Source: Front Nutr. 2026 Apr 21;13:1780653. doi: 10.3389/fnut.2026.1780653 (PMC13138985; doi:10.3389/fnut.2026.1780653)

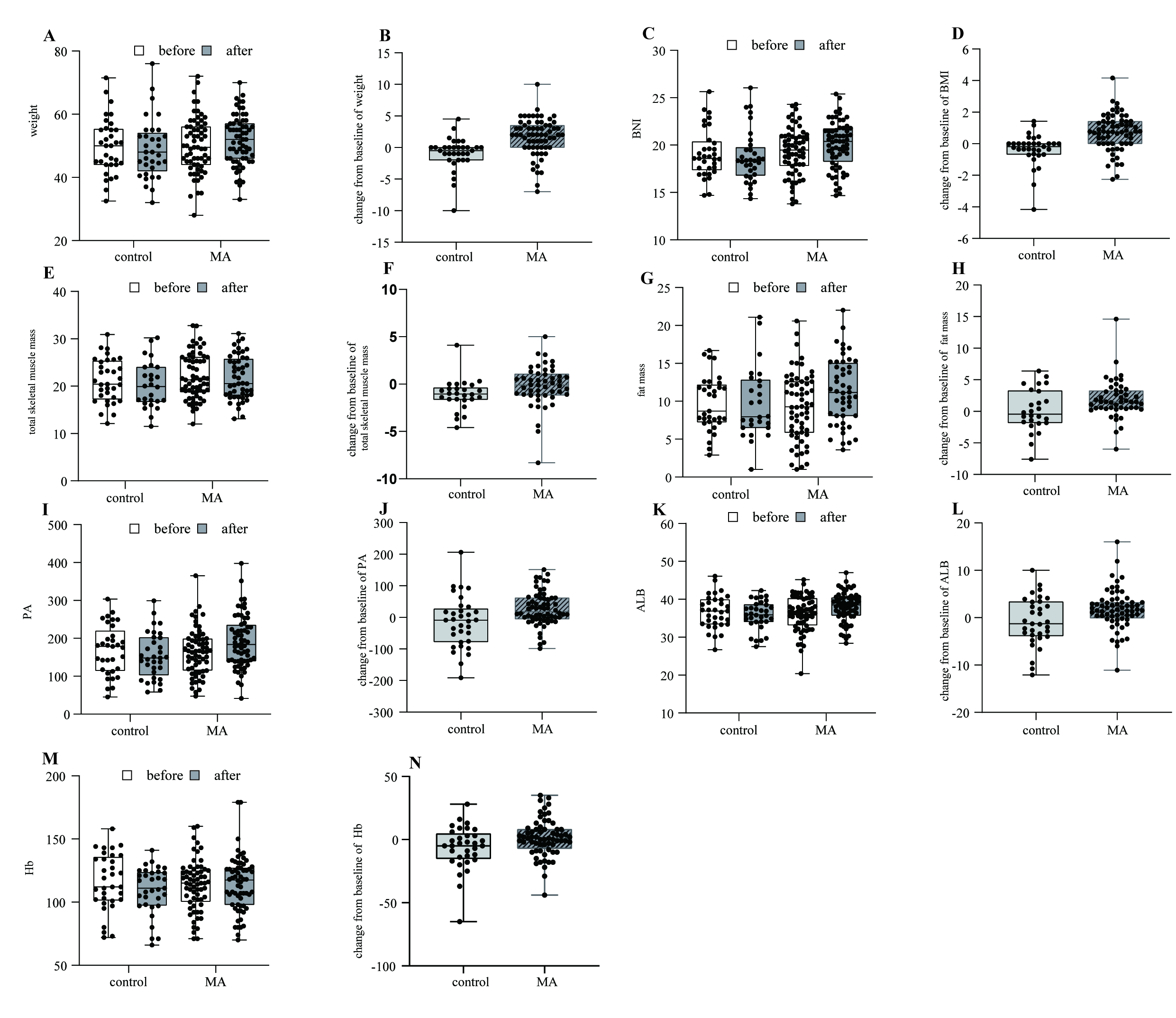

Supplement: Supplementary file 1 [file Image_1.tif]

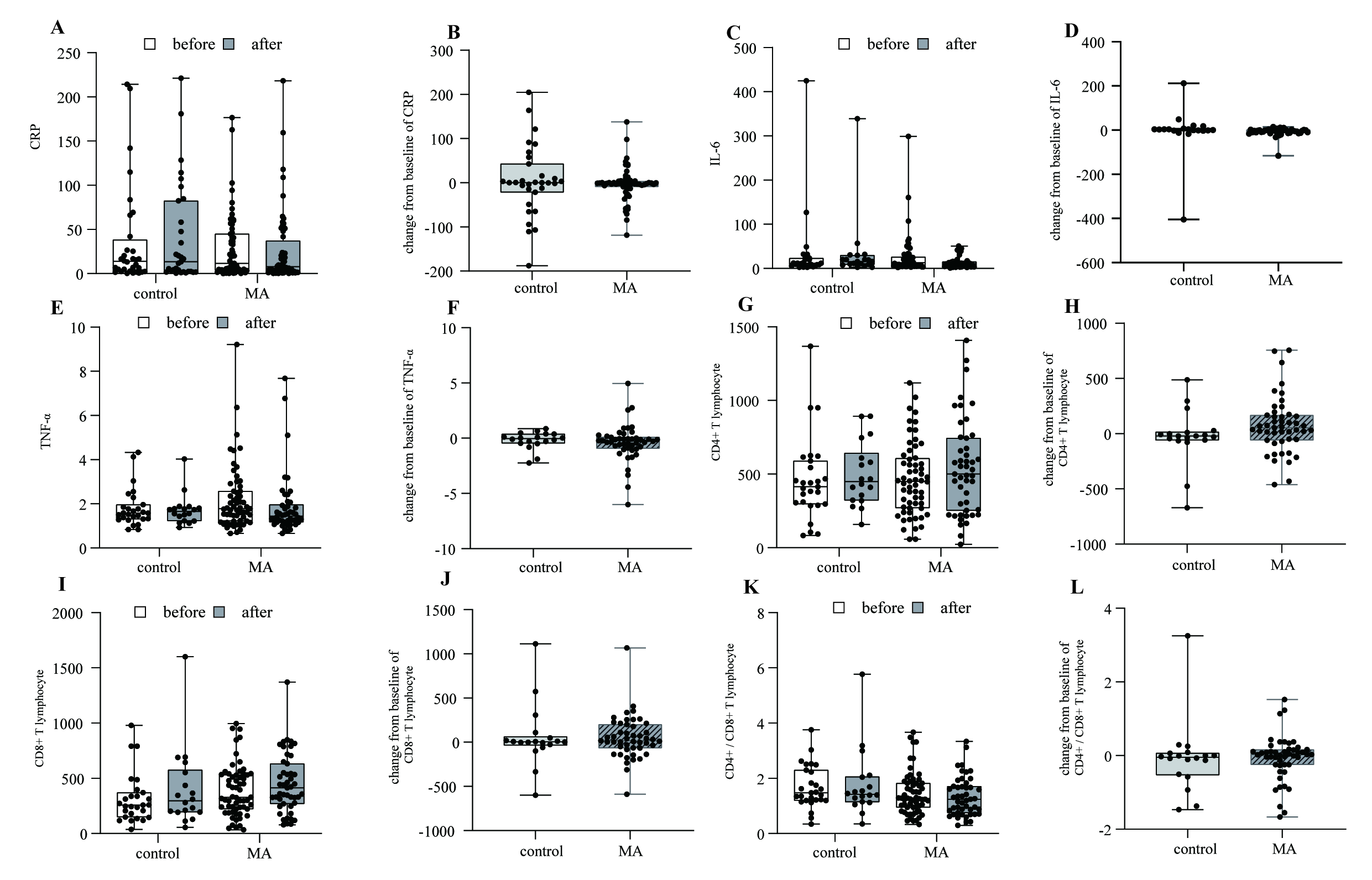

Supplement: Supplementary file 2 [file Image_2.tif]

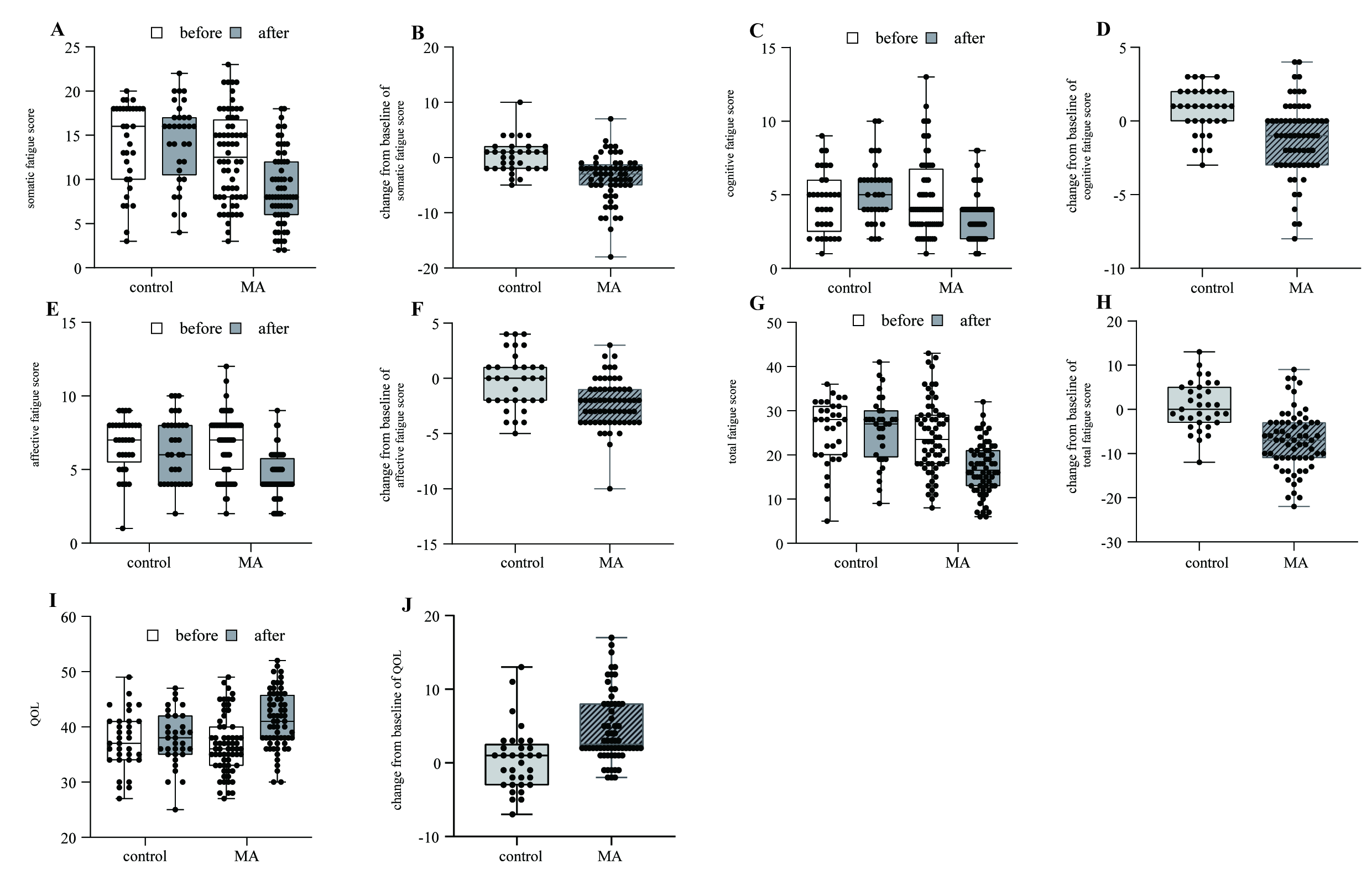

Supplement: Supplementary file 3 [file Image_3.tif]
