# Supplementary material for: Megestrol acetate in the management of cancer cachexia: a prospective quasi-experimental study focusing on body composition and patient-reported outcomes
Source: Front Nutr. 2026 Apr 21;13:1780653. doi: 10.3389/fnut.2026.1780653 (PMC13138985; doi:10.3389/fnut.2026.1780653)
Supplement: Supplementary file 4 [file Supplementary_file_1.docx]

Supplementary Figure legend

Supplementary Fig. S1 Individual changes and distribution of nutritional indicators before and after intervention. Box plots showing individual values, median (center line), interquartile range (box), and whiskers extending to the minimum and maximum for each group at baseline and after 2 months. Dots represent individual patients. (A, B) Weight, (C, D) BMI, (E, F) Skeletal muscle mass, (G, H) Fat mass, (I, J) PA, (K, L) ALB, (M, N) Hb. This figure complements the GEE–based estimated least squares means presented in Fig. 2 by providing detailed visualization of data distribution and individual variability.

### Supplementary Fig. S2 Individual changes and distribution of inflammatory and immune parameters before and after intervention. Box plots showing individual values, median (center line), interquartile range (box), and whiskers extending to the minimum and maximum for each group at baseline and after 2 months. Dots represent individual patients. (A, B) CRP, (C, D) IIL-6, (E, F) TNF-a, (G, H) CD4+ T cell count, (I, J) CD8+ T cell count, (K, L) CD4+/CD8+ T cell ratio. This figure complements the GEE-based estimated marginal means presented in Fig. 3.

### Supplementary Fig. S3 Individual changes and distribution of fatigue scores and quality of life before and after intervention. Box plots showing individual values, median (center line), interquartile range (box), and whiskers extending to the minimum and maximum for each group at baseline and after 2 months. Dots represent individual patients. (A, B) Somatic fatigue score, (C, D) Cognitive fatigue score, (E, F) Affective fatigue score, (G, H) Total fatigue score, (I, J) Quality of life (QOL) score. This figure complements the GEE-based estimated marginal means presented in Fig. 4.
